# Effect of monocular sensitivity on binocular summation of luminance-modulated flicker

**DOI:** 10.1371/journal.pone.0280785

**Published:** 2023-01-24

**Authors:** C. Vijay Reena Durai, Amithavikram R. Hathibelagal, Marisa Rodriguez-Carmona, John L. Barbur, Shrikant R. Bharadwaj

**Affiliations:** 1 School of Health Sciences, The Henry Wellcome Laboratories for Vision Science, Centre for Applied Vision Research, University of London, London, United Kingdom; 2 Brien Holden Institute of Optometry and Vision Sciences, L V Prasad Eye Institute, Hyderabad, Telangana, India; 3 Prof. Brien Holden Eye Research Centre, Hyderabad Eye Research Foundation, L V Prasad Eye Institute, Hyderabad, Telangana, India; Justus Liebig Universitat Giessen, GERMANY

## Abstract

Binocular summation of luminance contrast signals in the spatial domain has been investigated in many studies, but less attention has been paid to the analogous interactions in the temporal domain. The present study determined the impact of monocular sensitivity on the binocular detection of luminance-modulated flickering stimuli. Binocular summation ratios (BSRs) were determined in 13 visually-normal adults for a range of monocular flicker modulation thresholds (FMTs), generated by changing stimulus size (7’– 60’) and luminance (mesopic and photopic). Monocular and binocular FMTs were measured at the point of regard and in each of the four quadrants at 5° eccentricity for each target size and luminance using the Flicker-Plus test. Monocular and binocular FMT’s increased with decreasing target size for all retinal locations (p<0.001), and were overall larger for mesopic than for photopic condition (p<0.001). BSRs for mesopic (mean±SD: 1.50±0.21) and photopic (1.60±0.24) stimuli were greater than unity (p<0.001), with the latter showing larger estimates than former (p<0.001). BSRs showed no significant trend across target sizes for both luminance conditions (p>0.12). The results demonstrate that the visual system successfully summates inputs from the two eyes to enhance flicker detection, independent of their absolute monocular detection thresholds. These findings may serve as a predictive baseline for further experiments designed to determine how other stimulus properties and interocular differences in monocular thresholds may affect the binocular perception of flicker.

## Introduction

Fusion of monocular retinal images into a binocular cyclopean percept enhances spatial, temporal and depth vision [1]. One such enhancement is the improvement in the detection of spatial and temporal signals when stimuli are viewed binocularly, relative to monocular viewing conditions. This binocular advantage in signal detection is typically quantified by taking the ratio of monocular (better eye) to binocular thresholds, i.e., the binocular summation ratio (BSR) [2–6]. While binocular summation of contrast detection in the spatial domain has been thoroughly investigated, analogous effects in the temporal domain have been studied less extensively [2, 7]. The available literature on this topic does report that flicker detection thresholds measured using sine wave stimuli of a given spatial and temporal frequency yield BSRs that are similar to what is found in spatial contrast summation experiments (i.e., BSRs = 1.4 to 2.0).

The present study is concerned with how our ability to detect flicker monocularly can influence its enhancement under binocular viewing conditions. Two independent lines of empirical evidence suggest the existence of such an influence. First, Baker et al.’s meta-analysis showed that BSRs for flicker detection decrease with increasing temporal frequency, albeit with large intersubject variability [2]. Second, Grigsby and Tsou observed the BSRs to increase with stimulus temporal frequency only in the far periphery (24° and 32° from fovea) but not in near-foveal locations (0° to 8°), even while the overall BSR values decrease with retinal eccentricity in their subjects [8]. The reduction in BSR with temporal frequency and retinal eccentricity may arise from the well-established drop in monocular flicker modulation thresholds (FMTs), notwithstanding the contradictory results of Grigsby and Tsou [8] and the influence of many other factors unrelated to the monocular FMTs [e.g., relative contribution of cone- and rod-photoreceptors and the naso-temporal asymmetry of FMTs to binocular summation of flicker in Grigsby and Tsou’s study (8)]. Unlike the inverse relation between BSR and monocular FMT inferred from the earlier point, a directly proportional relation between the two may be expected from neurophysiology and signal detection theory [9]. Neurophysiologically, binocularly tuned neurons have been shown to play a greater role in signal detection than monocularly-tuned neurons in instances where the monocular thresholds are overall poor [9]. From a signal detection standpoint, subjects with lower monocular thresholds may be operating close to the limits of physiological performance, with little room for further improvement under binocular viewing [10, 11].

To address this gap in the literature, the present study systematically investigated the dependence of binocular summation of FMTs on the subject’s absolute monocular threshold for flicker detection. The study employed two methods to produce a range of different monocular FMTs for similar retinal locations near the fovea. In the first method, the size of the flickering stimulus was altered to vary the monocular FMTs under photopic cone-enhanced conditions based on a previous demonstration that the monocular FMTs increase with a reduction in angular subtense of flickering stimuli [12, 13]. In the second method, FMTs were re-measured across the same range of target sizes under mesopic stimulus conditions, which, again, has been shown to increase the monocular FMTs relative to photopic viewing [13]. The study hypothesized that, if BSR was directly proportional to monocular FMT, then BSRs for flicker would be higher for smaller stimuli under mesopic conditions than for larger stimuli under photopic conditions. The results will be reversed if BSR was inversely proportional to monocular FMT. Overall, these results of this study may provide new insights into how our ability to detect flicker with one eye may influence the binocular perception of flicker. These data may form the basis for future investigations on how binocular summation of flicker is affected by overall or interocular differences in sensitivity between the two eyes that can arise as a result of changes in the neurosensory retina in one or both monocular visual pathways (e.g., differential light absorption between the two eyes that can alter retinal sensitivity to contrast in the affected eye and also result in time delays between the signals from the two eyes [14, 15]).

## Methodology

Thirteen subjects [mean ± 1SD age: 26 ± 2years; 3 males and 10 females], naïve to the study protocol, were enrolled for this study from amongst the staff and students of the L V Prasad Eye Institute (LVPEI), Hyderabad, India. The study adhered to the tenets of the Declaration of Helsinki and was approved by the Institute Research Board of LVPEI and the Senate Research Committee of City, University of London. A written informed consent was obtained from each study participant. Inclusion criteria required each participant to be free from any sensory and motor ocular pathology following a comprehensive eye examination. All study participants had best corrected, high contrast visual acuity of 0.0 logMAR (20/20) or better.

**Fig 1:**
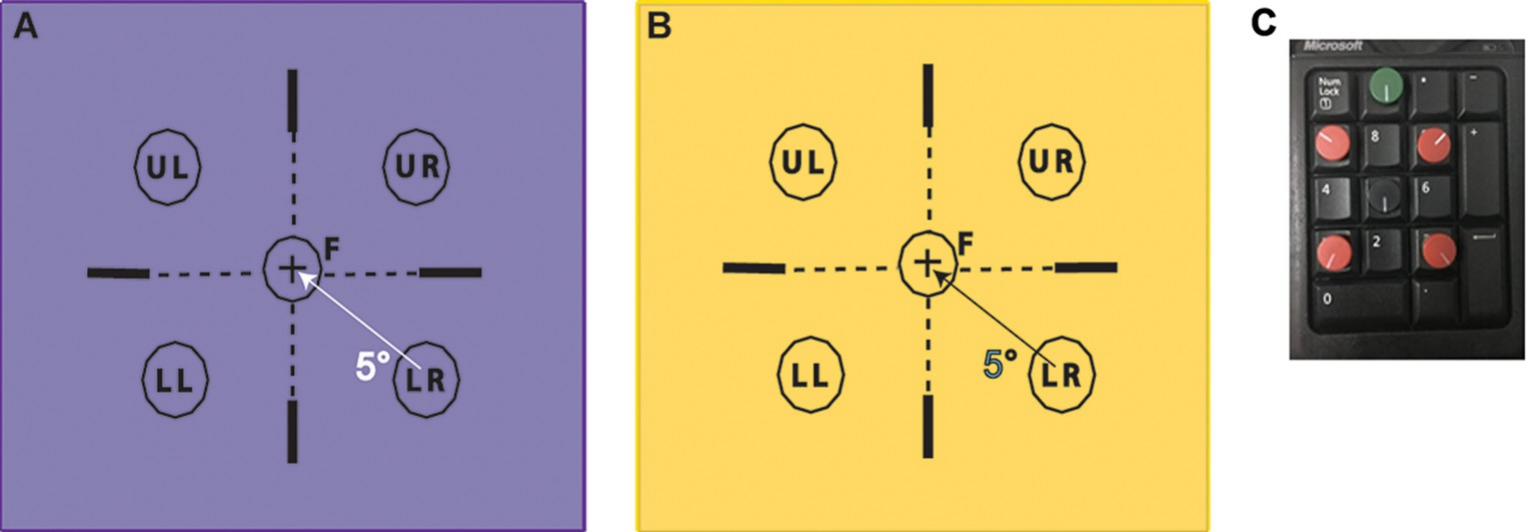
Schematic representation of the stimulus display for mesopic (panel A) and photopic (panel B) conditions used in this study.

The two approaches employed in this study to vary the absolute value of monocular FMTs were executed using the Flicker-plus module in the Advanced Vision and Optometric Tests setup (AVOT, City Occupational Ltd., UK). Here, the flicker stimulus was a uniform circular patch presented on a fully calibrated LCD computer monitor (1920 X 1200pixels resolution: 60Hz). Each primary colour of the stimulus display was calibrated for spectral radiance using a Minolta CS 2000 tele spectroradiometer (Konica Minolta, Basildon UK) and for luminance over the entire 10-bit dynamic range using the LMT 1009 photometer (LMT, Berlin, Germany) using the LUMCAL calibration program supplied by City Occupational Ltd., London, UK. The relative spectral composition of the test and background during the presentation of the stimulus remained unchanged. All subjects viewed the visual display from 1m (Fig 1). The size of the flicker stimulus was set to 7’, 15’, 30’, 45’ and 60’ to systematically vary monocular FMTs to execute the first approach. Pilot data showed that the measured FMTs did not vary significantly for target sizes beyond 60’, thus helping us determine the range of target sizes to be employed in this study. FMTs for all target sizes were measured in both mesopic and photopic stimulus conditions, with the flicker stimulus having parameters detailed in Table 1 [13]. Three full cycles of sinusoidal temporal modulation at 5Hz were employed for the mesopic condition to favour rod photoreceptors and 5 full cycles of square-wave modulation at 15Hz was employed for the photopic stimulus condition, to favour cones. The mean luminance of the flickering stimulus remained unchanged during the entire experiment, and it was equal to that of the uniform background field. All the stimuli started from 10% contrast modulation and the stimulus presentation times employed were selected to ensure an integer number of cycles were presented for both photopic and mesopic modulation conditions (Table 1). The low background luminance needed for the mesopic condition was achieved by viewing the display through spectrally calibrated, ‘neutral density’ glasses with a nominal optical density of 1 log unit. As a result, the temporal modulation contrast of the flickering stimulus remained unaffected by pre-receptoral filters in the eye with no contribution from chromatic mechanisms.

**Table 1:** Optimized parameters for mesopic and photopic condition used in the study.

Parameters	Mesopic	Photopic
Temporal frequency (Hz)	5	15
Luminance (cd/m^2^)	0.5	24
Eccentricity (degrees)	5	5
CIE (x,y) coordinates	0.18, 0.089	0.58, 0.36
Scotopic/Photopic ratio	9	0.9
Duration (ms)	600	334

Monocular FMT of each eye and binocular FMTs were measured twice in each subject for every combination of target size and the two luminance conditions. Every subject therefore participated in a total of 60 test conditions in randomized testing order [5 target sizes x 2 luminance levels (photopic, mesopic) x 3 viewing conditions (monocular RE, monocular LE, binocular) x 2 repetitions = 60 runs]. Each test run involved five, randomly-interleaved adaptive staircases, one for each of the five stimulus locations [i.e., centrally at the point of regard and diagonally at an eccentricity of 5° in each of the four quadrants—lower-right (LR), lower-left (LL), upper-right (UR) and upper-left (UL) in spatiotopic coordinates (Fig 1)]. The subject’s task was to identify the location of the stimulus on each presentation. Based on their response, the stimulus contrast was increased or decreased until a total of nine reversals were obtained for each test location. The average contrast of the last six reversals for staircase was considered as the FMT of the tested location.

Before the start of the experiment, a learning module was given for the subject to become acquainted with the test protocol. Once the test was initiated for a given stimulus condition, an auditory cue and a briefly presented central cross and square prompted the subject to attend to the center of the screen. The disappearance of the fixation was then followed 500ms later by a flicker stimulus which appeared at one of the five, randomly selected locations (Fig 1). The subjects were instructed to attend to the center of the screen and they were provided with a numeric keypad (Fig 1C) that had five buttons arranged to mimic the five stimulus locations on the visual display (Fig 1). The subjects were asked to respond by pressing one of the buttons (red, in Fig 1C) according to the location of the stimulus. A separate button (green, in Fig 1C), placed at the top of the numeric keypad was allocated for use when the subject was completely unaware of any stimulus presentation (Fig 1C). When this button was pressed, the program assigned the response randomly to one of the five target locations. A response was considered to be correct if the subject chose the stimulus location of the presentation appropriately. The staircases employed a five-alternative, one-up, two-down response procedure to arrive at the threshold. Each staircase employed nine reversals and the subject’s threshold was computed by averaging the last six reversals [13, 16]. The use of double guides and a dynamic fixation target at the beginning of each presentation with short stimulus presentation times made it easier for the subject to attend to the center of the screen and avoid eye-movements during the stimulus (Fig 1). Eye movements during the experiment were not explicitly monitored. Subjects were reminded throughout the test to attend to the center of the screen upon activation of the fixation cue.

A fixation cross (F) at the center to maintain the fixation of the subjects, prompts before the occurrence of the stimulus. Stimulus locations were denoted as UL-Upper Left, UR-Upper Right, LL-Lower Left, LR-Lower Right and C- central position. Other details of the stimuli are noted in Table 1. The background colour in panels a and b may not represent the actual colour in the test but are close enough to the original experiment. Panel c is the numeric keypad used to collect responses from subjects in the study.

Subjects wore their optimal spectacle correction, when required, during the experiment. An opaque occluder was used to cover one eye in monocular experiments and order of testing was randomised. The entire experiment took ~8 hours to complete on a given subject and this was conducted on different days in several ½ hour to 1-hour blocks with breaks between test conditions to minimise fatigue.

### Data analysis

**Fig 2:**
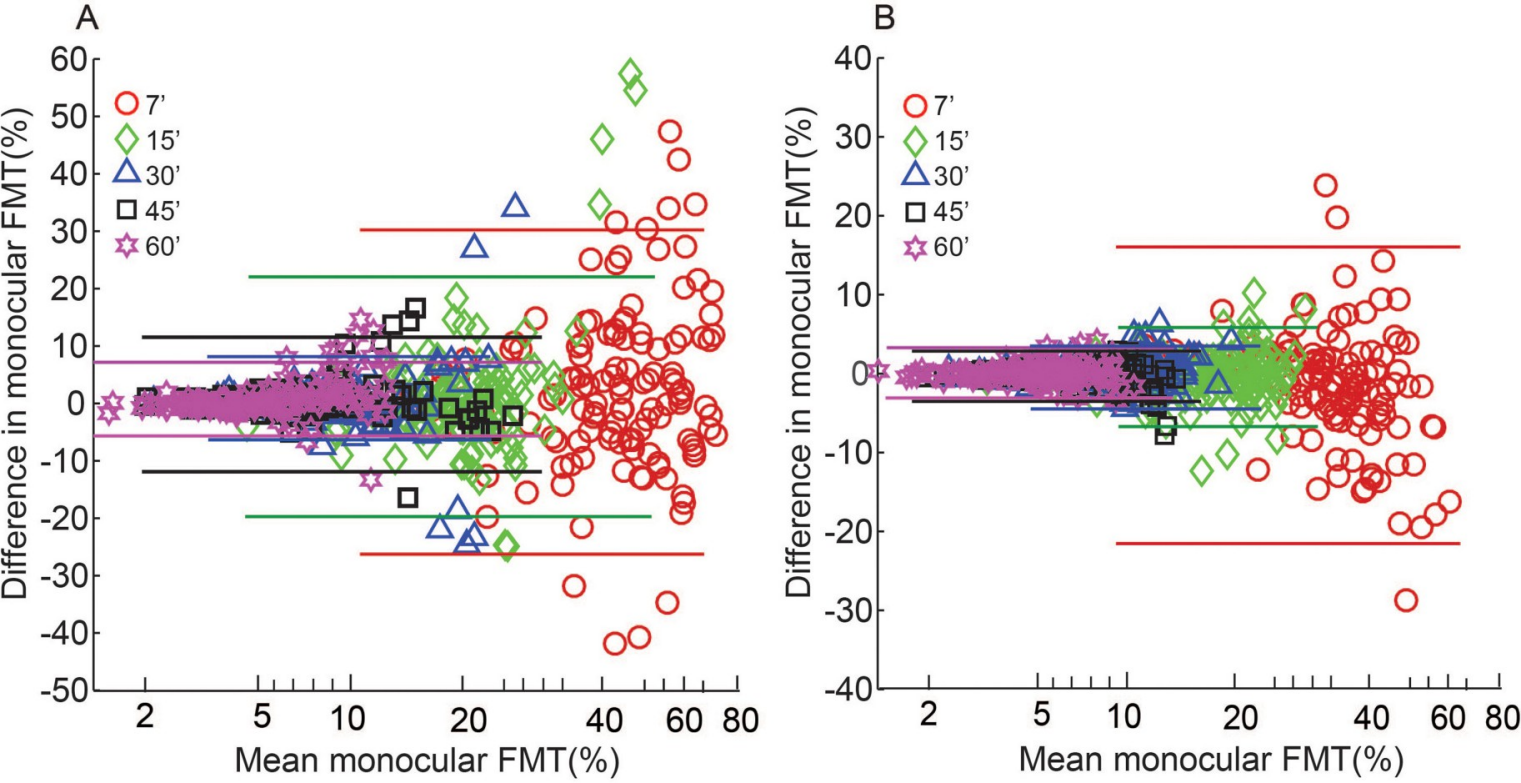
Bland-Altman type plots of intersession difference. Monocular FMTs are plotted as a function of the corresponding mean FMT for different target sizes under mesopic (panel A) and photopic (panel B) viewing conditions. Different colored solid horizontal lines indicate ±95% limits of agreement in FMT between sessions for the respective target sizes, respectively.

**Fig 3:**
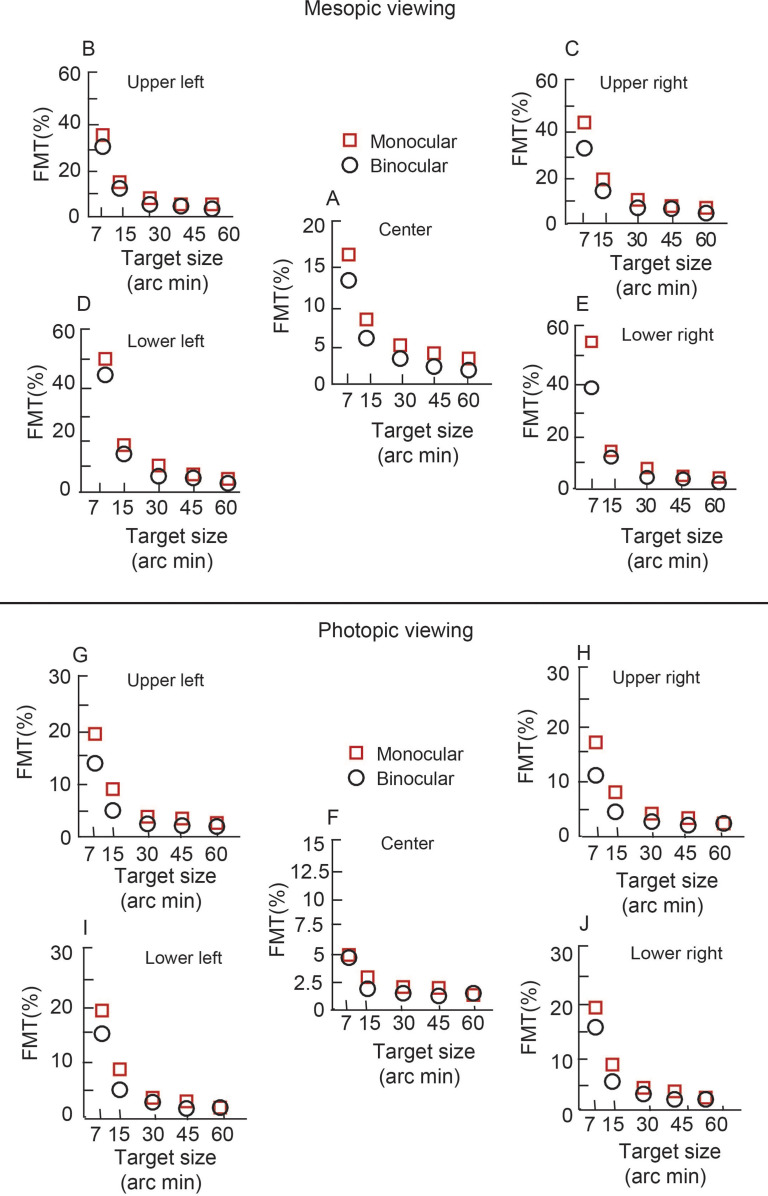
Representation of better of the two eyes’ monocular FMTs and binocular FMTs. Mesopic (top) and photopic viewing (bottom) conditions for an individual subject at the point of regard and in each of the four quadrants plotted. Each data point represents the average FMT of the two runs for that target location. Ordinates for panels b to e and g to j are different from panels a and f, respectively. Ordinates are also different between mesopic and photopic conditions.

**Fig 4:**
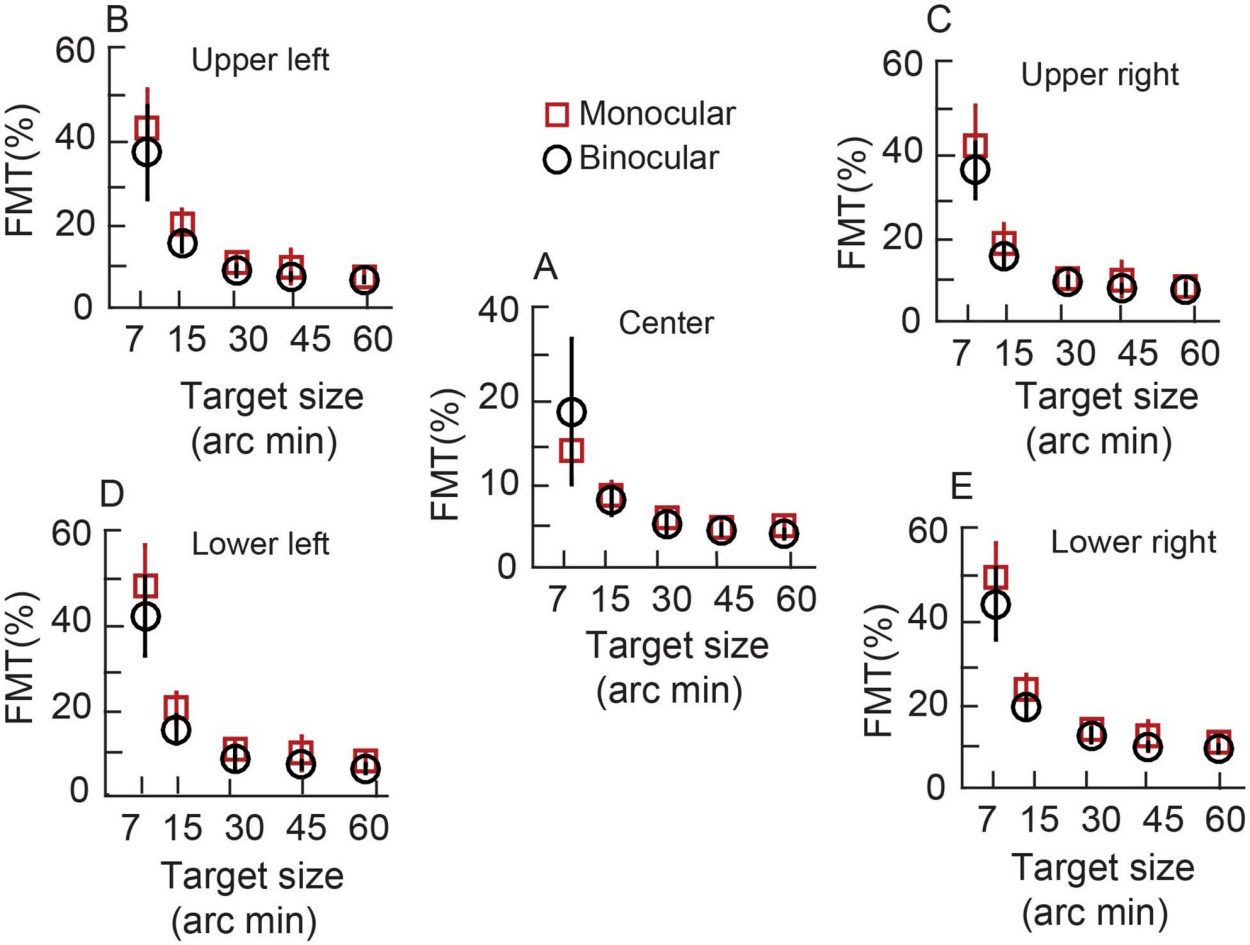
Mean (±1SD) better of the two eyes’ monocular FMTs and binocular flicker thresholds across 13 subjects plotted for mesopic condition as a function of target size. All other details are same as Fig 3.

**Fig 5:**
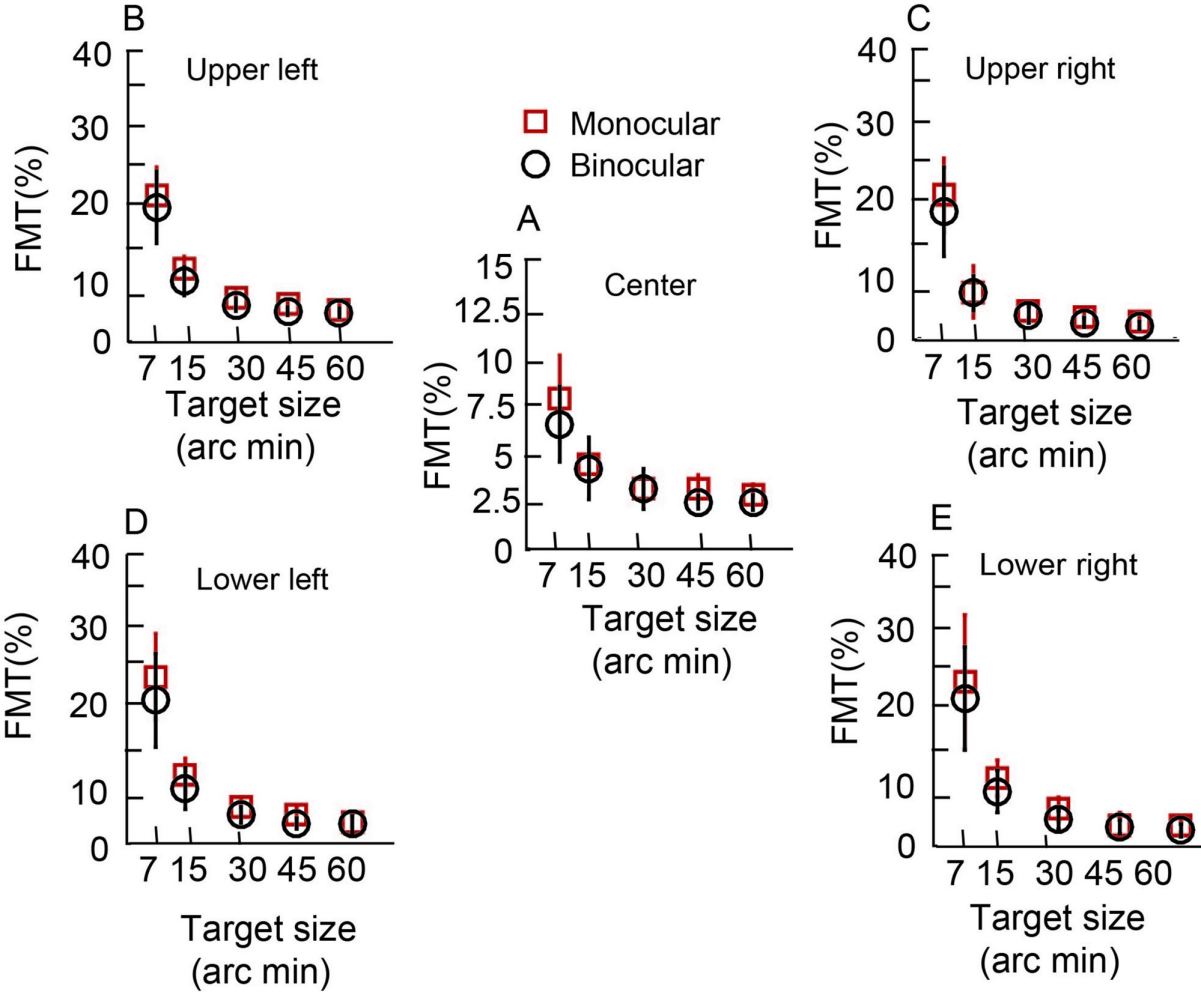
Mean (±1SD) better of the two eyes’ monocular FMTs and binocular flicker thresholds across 13 subjects plotted for photopic condition as a function of target size. All other details are same as Fig 3.

**Fig 6:**
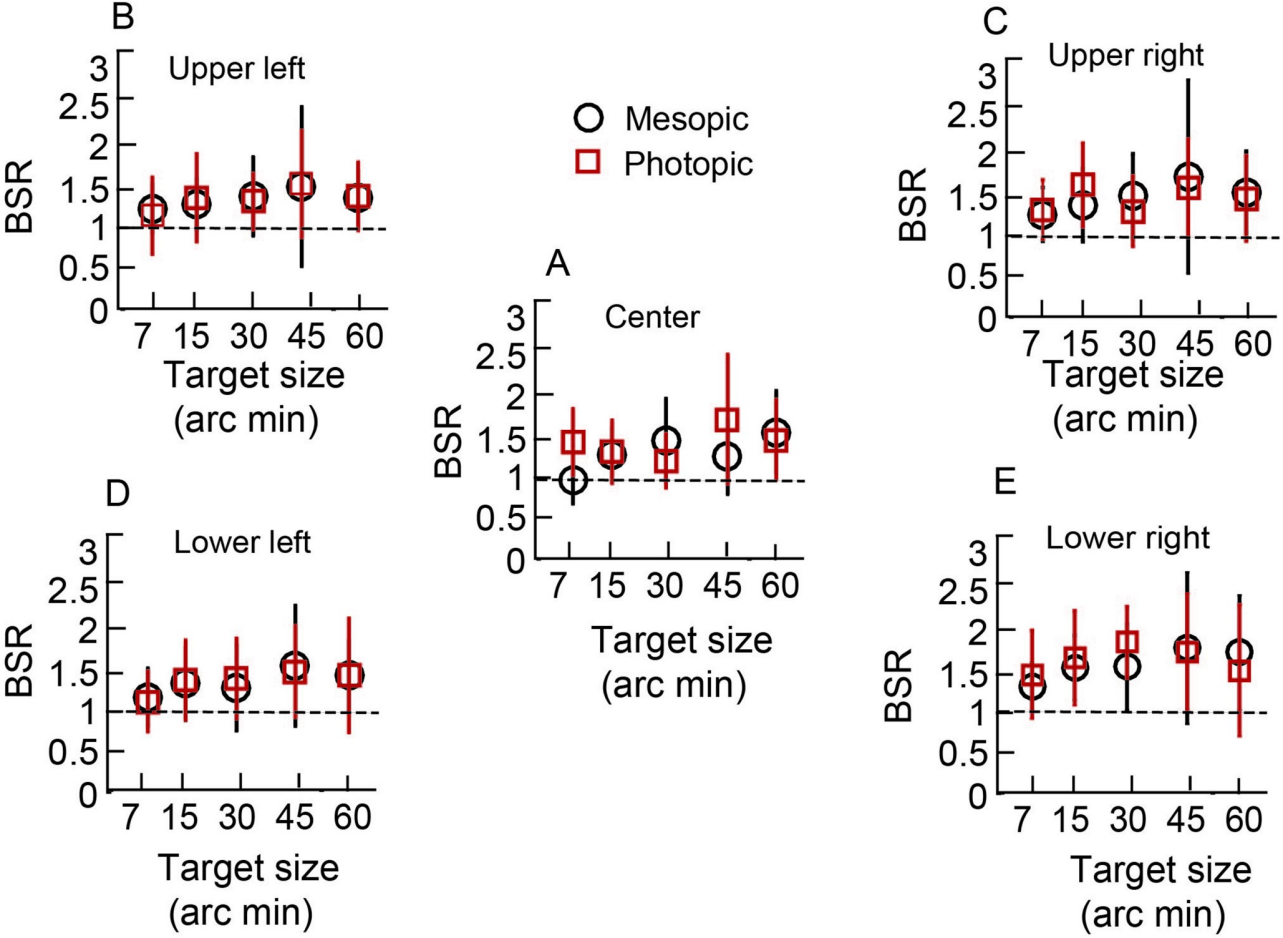
Binocular summation ratio. Mean (±1SD) binocular summation ratio (BSR) of FMTs as a function of target size for the mesopic and photopic stimulus conditions at point of regard and the four parafoveal locations. Horizontal dashed line in each panel indicates no binocular summation.

Data analysis was performed with Matlab^®^ R2016a (Mathworks, Natick, USA) and IBM SPSS^®^ Statistics 20.0 (SPSS, Chicago, USA). As observed earlier [6], the monocular and binocular FMTs obtained from the four parafoveal target locations were not significantly different from each other and hence they were averaged for ease of statistical analysis. The data measured for each parafoveal target location are however shown separately in Figs 3–6. Any trend in the monocular FMTs or interocular differences in monocular FMTs or BSRs with target size or viewing condition are meaningful only when the measures of FMT are repeatable across trial runs. The two repeated runs of monocular FMTs obtained for each test condition in this study was therefore used to curate the data for performance reliability. Fig 2 shows the Bland-Altman type plot of intersession variability in monocular FMTs plotted as a function of the mean monocular FMT for different target sizes under mesopic (panel A) and photopic (panel B) viewing conditions (See S1_datasheet.xls in S1 Data for raw data and FMT_BSR_Analysis.m for Matlab code plotting the raw data). While there was no mean difference in the monocular FMTs between the two sessions for overall mesopic [mean difference ±95% limits of agreement (LOA): (0.86±1.47%) and photopic (0.16±1.45%) conditions, the 7’ stimulus showed the largest intersession variability in FMTs (mean±1SD for mesopic: 3.26±2.9%; photopic: 1.68±2.3%), relative to other target sizes (mesopic: 1.46±0.84%; photopic 0.42±0.65%, for all) (Fig 2). ±95% LOA for each target size was estimated and the points lying outside these limits were considered as outliers, reflecting unreliable responses, and removed from the final analysis. All these data points were also identified as outliers in the Grubb’s test [17]. Removal of data point of one eye also resulted in the removal of the fellow eye and binocular data point for that target size to avoid artefacts in the results. In this process, a total of 5, 9, 10, 7 and 12 data points from both eyes put together were triaged for target sizes 7’, 15’, 30’, 45’ and 60’, respectively, in the mesopic condition and 10, 6, 6, 6 and 11 data points from both eyes put together were triaged for 7’, 15’, 30’, 45’ and 60’, respectively, in photopic condition. BSRs were calculated for each stimulus combination as the ratio of the monocular FMT for the best eye to the corresponding binocular threshold. Alternate methods for calculating the BSR such as averaging the two monocular outputs or normalizing the components of the binocular input to their respective monocular thresholds was not followed, for the former technique is known to overestimate BSRs and the latter technique is meaningful only in situations where the two monocular thresholds exhibit significant interocular differences [2].

Kolmogorov-Smirnov test indicated that the monocular and binocular FMTs and the corresponding BSRs were normally distributed, and hence parametric statistics were employed for data analyses. To determine the effect of target size, location (point of regard vs. parafoveal) and viewing condition (monocular vs. binocular) on the FMTs, a 3-factor, repeated measures analysis of variance (RM-ANOVA) was performed separately for the mesopic and photopic conditions. Similarly, to determine the impact of target size and target location on the BSRs, a 2-factor RM-ANOVA was performed separately for the mesopic and photopic conditions. Tukey post-hoc analysis was subsequently performed for individual pairwise comparisons of target size, wherever applicable. P≤0.05 was considered statistically significant. Effect size was quantified using the partial Eta-squared (η_p_^2^) value [18].

## Results

### Variation of monocular and binocular FMT in one representative subject

Data of monocular and binocular FMTs were collected successfully from all 13 subjects who participated in the study (See S1_datasheet_WO.xls in S1 Data for raw data and FMT_BSR_Analysis.m for Matlab code plotting the raw data). Fig 3 shows changes in the better of the two eyes’ monocular FMT and binocular FMTs plotted as a function of target size under mesopic (top) and photopic (bottom) viewing conditions for a representative subject that participated in the study. The data clearly showed a significant drop in the monocular and binocular FMTs with increasing target size, with the FMTs being overall lower for photopic than mesopic viewing conditions, indicating relatively higher sensitivity for flicker detection in the former than latter condition (Fig 3). The binocular FMTs were also smaller than the corresponding monocular FMTs, indicating improved flicker detection in the former than latter viewing condition (Fig 3).

### Variation in monocular and binocular FMTs across the entire cohort

Like the data of the representative subject, the cohort-level mean (±1SD) better of the two eyes’ monocular FMT and binocular FMTs decreased with increasing target size across target location for both mesopic (Fig 4) and photopic (Fig 5) viewing conditions (See S1_datasheet_WO.xls in S1 Data for raw data and FMT_BSR_Analysis.m for Matlab code plotting the raw data). The mean monocular FMTs were also larger than their binocular counterparts across all viewing conditions for both the mesopic and photopic stimulus conditions (Figs 4 and 5). The 3-factor RM-ANOVA (target size x target location x viewing condition) for FMTs obtained in the mesopic stimulus condition showed a statistically significant main effect of target size [F(4,373) = 279, p = 0.001, η_p_^2^ = 0.82)], target location [F(1,318) = 141, p = 0.001, η_p_^2^ = 0.37)] and viewing condition [F(1,238) = 31, p = 0.001, η_p_^2^ = 0.11)]. There was also a statistically significant interaction between the three factors [target size x target location: F(4,238) = 38.39, p = 0.001; target size x viewing condition: F(4,238) = 2.6, p = 0.03; target location x viewing condition: F(4,238) = 14.9, p = 0.001; target size x target location x viewing condition: F(4,277) = 2, p = 0.04], indicating that monocular and binocular FMTs did not vary uniformly in relation to the factors investigated in this study. Pairwise comparisons with the Tukey HSD test for target size showed that the FMTs for the 7’,15’ and 30’ targets were significantly different from each other (p = 0.001) and also from all other target sizes (p = 0.001). None of the other pairwise comparisons were statistically significant (p≥0.52).

Three-factor RM-ANOVA (target size x target location x viewing condition) for FMTs in the photopic stimulus condition showed trends like the mesopic data. There was a statistically significant main effect of target size [F(4,240) = 102, p = 0.001, η_p_^2^ = 0.62)], target location [F(1,240) = 82, p = 0.001, η_p_^2^ = 0.24)] and viewing condition [F(1,240) = 14, p = 0.001, η_p_^2^ = 0.05)]. There was a statistically significant interaction between all the factors [target size x retinal location: F(4,240) = 86, p = 0.001; retinal location x viewing condition: F(1,240) = 6, p = 0.02; target size and viewing condition [F(4,240) = 5.6, p = 0.001; target size x retinal location x viewing condition: F(4,240) = 86, p = 0.01]. Like the mesopic stimulus condition, pairwise comparison for the 7’ and 15’ targets were significantly different from each other (p = 0.001) and from all other target sizes (p = 0.01). None of the other pairwise comparisons were statistically significant (p≥0.38). These results confirm previous findings that the monocular and binocular FMTs vary systematically with the size and location of the flickering stimulus–the highest sensitivity is measured with large stimuli in central vision [13].

Even while the overall trend of FMTs with target size and target location were similar in the two conditions, there were quantitative differences in the absolute values of monocular and binocular FMTs between the two conditions, reflecting differences in the processing of flicker information by the two luminance conditions (Figs 4 and 5). The change in monocular FMTs from the smallest to the largest target size was similar for both conditions for central and parafoveal targets (Table 2). The fold-change in Table 2 was obtained by dividing the FMT for the smallest by the largest target size. Larger fold-change indicated greater reduction of FMT with a decrease in target size. The corresponding change in binocular FMTs with target size was larger for the mesopic background than for the photopic, more so for center point of regard targets than for parafoveal targets (Table 2). These results confirm the previous findings of Hathibelagal et al. [13] that the visual system is more sensitive at detecting flicker under photopic than under mesopic conditions for both monocular and binocular viewing conditions and that the former is relatively more immune to target size variations than the latter.

**Table 2:** Mean (±95% CI) of fold-change in monocular and binocular FMTs from smallest (7’) to largest (60’) target size obtained for mesopic and photopic conditions for central and parafoveal target locations.

	Background type	Location	Fold-change	±95% CI
Mesopic	Monocular	Central	4.85	4.1–5.6
		Parafoveal	9.61	8.3–10.92
	Binocular	Central	8.95	5.97–11.93
		Parafoveal	12.77	10.55–14.99
Photopic	Monocular	Central	4.29	3.67–4.91
		Parafoveal	8.84	7.68–10
	Binocular	Central	4.52	3.73–5.31
		Parafoveal	10.12	8.02–12.22

### Binocular summation ratios (BSRs) for flicker perception

Fig 6A–6D show data of BSRs plotted as a function of target size for point of regard and parafoveal locations for photopic and mesopic viewing conditions (See S1_datasheet_WO.xls in S1 Data for raw data and FMT_BSR_Analysis.m for Matlab code plotting the raw data). The mean BSR across all testing conditions were greater than unity, indicating summation of monocular FMTs from the two eyes under binocular viewing conditions (Fig 6). The BSR trends were, however, slightly different for mesopic and photopic conditions–in general, the data for mesopic stimuli showed a greater increase in BSR with increasing target size for both point of regard and parafoveal locations whilst this effect was less obvious for photopic stimuli (Fig 6). These qualitative trends did not however withstand statistical significance testing. Three-factor RM-ANOVA (target size x target location x luminance type) for BSRs showed a marginally statistically significant main effect of only target location [F(4,240) = 3.94, p = 0.04, η_p_^2^ = 0.01)], while target size [F(1,240) = 2.13, p = 0.07, η_p_^2^ = 0.03)] and stimulus luminance [F(1,240) = 20, p = 0.77, η_p_^2^≤0.01)] were not statistically significant (Fig 6). There was no statistically significant interaction between the three factors [target size x luminance: F(4,240) = 2.24, p = 0.06, η_p_^2^ = 0.03; target location x luminance: F(1,240) = 0.51, p = 0.47, η_p_^2^ = 0.002; target size and target location [F(4,240) = 0.74, p = 0.56, η_p_^2^ = 0.012]; and target size x target location x luminance: F(4,240) = 1.0, p = 0.40, η_p_^2^ = 0.01. The pairwise comparison of the BSRs across all the quadrants in both mesopic and photopic conditions for the 7’ and 45’ targets were significantly different from each other (p = 0.008) and from the 60’ size (p = 0.028). None of the other pairwise comparisons were statistically significant (p≥0.77). These results clearly indicate that binocular viewing yields improved flicker detection thresholds, relative to monocular viewing, at all stimulus locations for both the mesopic and photopic stimulus conditions.

### Interocular variability of FMTs

**Fig 7:**
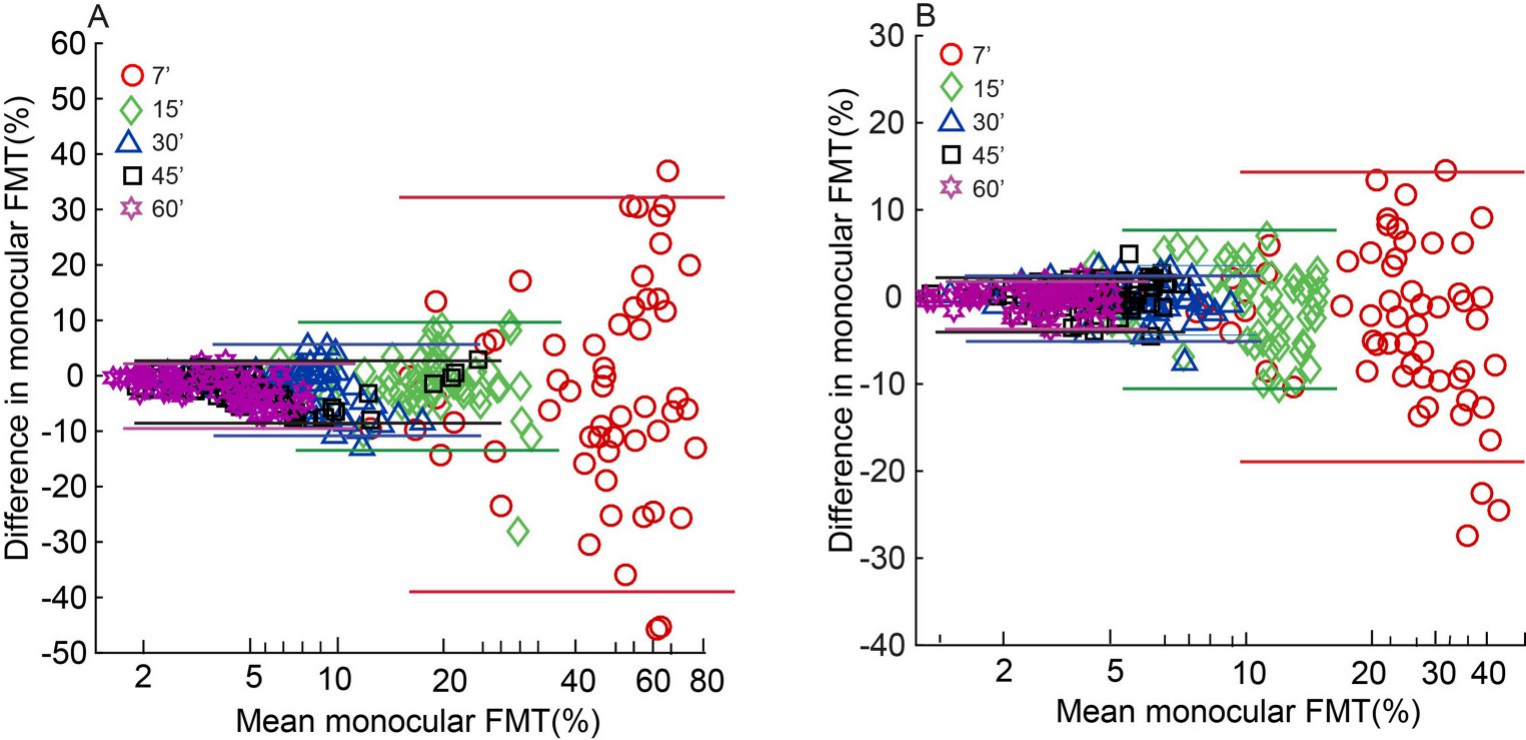
Bland-Altman plots of interocular difference in monocular FMTs plotted as a function of the corresponding mean FMT for the mesopic (panel A) and photopic viewing conditions (panel B). All other details are same as the Bland-Altman plots shown in Fig 2.

Even while monocular FMTs were expected to be similar in the two eyes of participants, significant interocular variability in these thresholds was observed under some testing conditions, as shown in the Bland-Altman plots in Fig 7 (See S1_datasheet_WO.xls in S1 Data for raw data and FMT_BSR_Analysis.m for Matlab code plotting the raw data). While there was no significant mean interocular difference in the monocular FMTs under mesopic (panel a) and photopic (panel b) viewing conditions (mean±95% LOA mesopic: 5.2±6.1% and photopic: 0.2±1.4%), the 7’ stimulus showed the largest interocular variability in FMTs (mesopic: 15.3±10.7%; photopic: 5.1±4.51%), relative to all other target sizes (mesopic: 3.1±2.7%; photopic: 1.4±1.12%, for all). The corresponding interquartile range of BSRs for data points lying outside the limits of agreement ranged from 0.98–1.37 for mesopic and 1.45–1.61 for photopic viewing conditions across all target sizes. These were comparable to the interquartile range of BSRs (1.15–1.75) for data points within the LOA, suggesting that naturally-occurring interocular differences in monocular FMT’s did not influence the magnitude of binocular flicker summation. No formal statistical analyses were run on this dataset, considering the small number of data points available in the large interocular difference dataset.

### Effect of number of repetitions of the psychophysical testing on binocular FMTs

**Fig 8:**
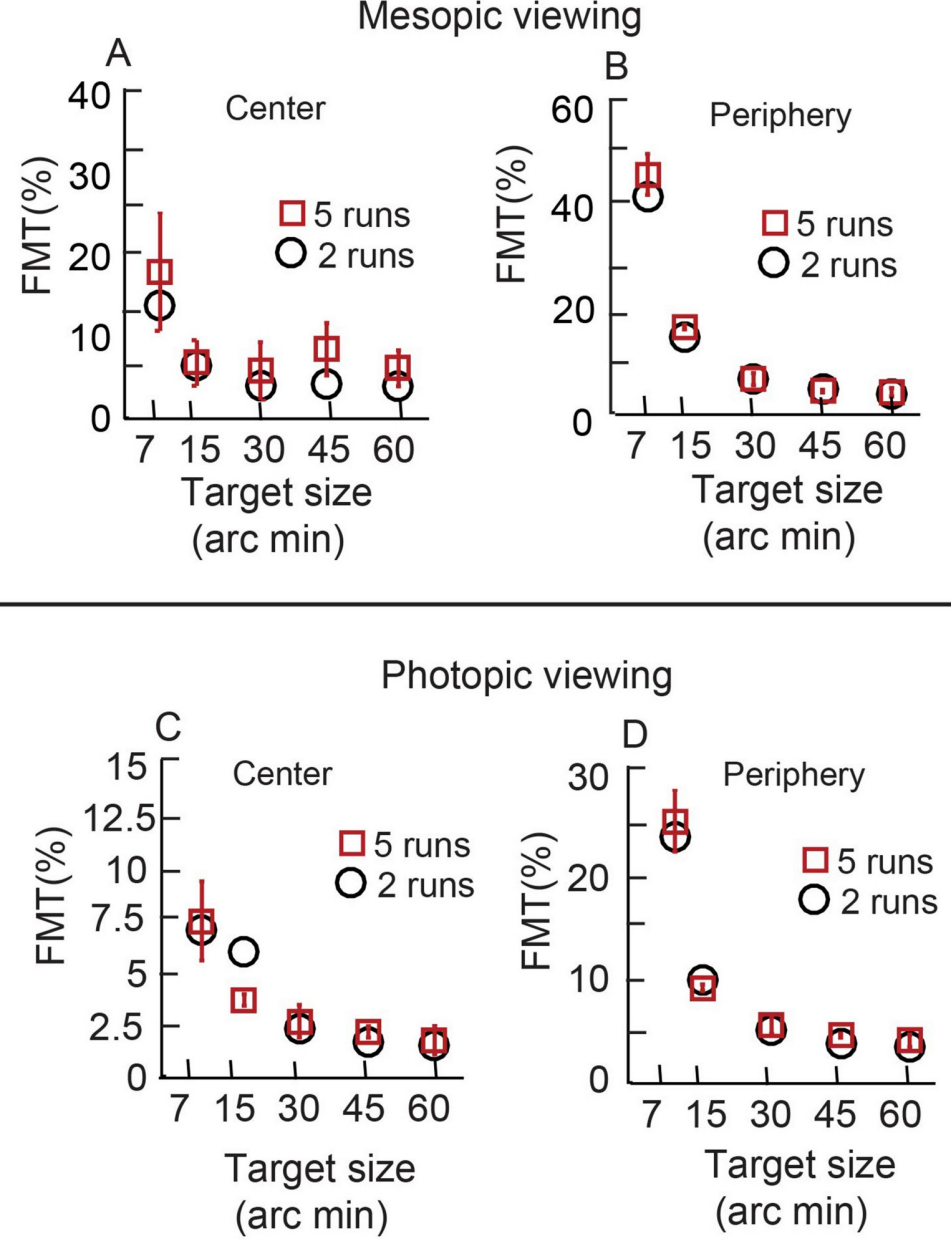
Representation of better of the two eyes’ monocular FMTs and binocular FMTs. Mean (±1SD) binocular FMTs obtained under mesopic (left panels; n = 1) and photopic (right panels; n = 3) viewing conditions for different stimulus sizes at the point of regard (panels A and B) and the four parafoveal locations (panels c and d) for 2 runs and 5 runs of the same experiment. Data from all four parafoveal locations are averaged in this figure.

FMTs for each combination of target size, location and viewing condition were obtained twice on each subject. While the usual number of repetitions in a typical psychophysical experiment will be larger than two, this was not possible on all subjects who participated in this study owing to the cumbersome nature of data collection. To determine if the trends observed in FMTs of this study varied with an increase in the number of repetitions, a control experiment was performed with five repetitions of the binocular FMTs for the different target sizes under mesopic viewing condition for one subject (Fig 8A and 8C) and photopic viewing condition for three subjects (Fig 8B and 8D) (See S1_datasheet.xls in S1 Data for raw data and FMT_BSR_Analysis.m for Matlab code plotting the raw data). The experimental paradigm was identical to the main experiment. The qualitative trends in the reduction of binocular FMTs with increasing target size was similar for the data obtained from two and five repetitions. Considering the limited sample size in this control experiment, statistical analysis was not performed in this dataset. Overall, the results indicated that the binocular FMT trends observed in the main experiment (Figs 3–6) reflected true changes in flicker perception capabilities with target size and not an artefact arising from the limited number of test repetitions.

## Discussion

This study tested the hypothesis that BSRs of temporally modulated luminance flicker varies inversely with absolute values of monocular FMTs. This hypothesis was tested by experimentally varying the monocular FMTs changing the angular subtense, luminance, chromaticity and temporal frequency properties of the flicker stimulus (Table 1). The monocular FMTs varied by a 4- to 16-fold range between the smallest and the largest target size and higher for mesopic than photopic viewing conditions, in accordance with previous literature (Table 2, Figs 3–5) [13, 16]. These trends were also repeatable across experimental runs (Fig 8), altogether indicating that the present experimental approaches were appropriate to test the study hypothesis. Binocular FMTs were found to be lower than their monocular counterparts in a vast majority of testing conditions in the present results, indicating successful binocular summation of the monocular signals (Figs 3–5). However, the BSRs did not show any specific trend across target sizes or luminance, indicating that binocular summation was largely independent of the absolute monocular FMTs (Fig 6). If any, there was only a weak qualitative trend for the BSRs to be higher under photopic (with lower monocular FMTs) than mesopic (with higher monocular FMTs) viewing conditions (Fig 6). Taken together, the present results do not support the hypothesis that the binocular summation of flicker is dependent on the absolute monocular flicker modulation thresholds. The visual system, thus, continues to achieve full binocular summation of flicker signals from the two eyes, irrespective of their monocular thresholds.

Overall, these results are in broad agreement with those of Grigsby and Tsou [8]. The invariance of BSR with monocular FMTs are in-line with their observation of no change in the BSRs of flicker for targets at the fovea and at 4° and 8° retinal eccentricity from fovea, despite a 10- to 12-fold change in monocular FMTs through variations in the temporal frequency of the stimulus [8]. The targets in the present study were located at the centre and at 5° retinal eccentricity and there was a 4- to 16-fold change in monocular FMTs between the two types of variations in the properties of the flicker stimulus (Table 1). Thus, even while the stimulus properties that lead to the variation in monocular thresholds may be different, the outcomes of the invariance of BSR with the underlying monocular thresholds remain as is. Grigsby and Tsou did, however, observe a reduction in BSR with an increasing retinal periphery, and this was attributed to the interocular differences in the monocular FMTs arising from naso-temporal asymmetries in flicker detection [8]. Much like the present study, an eccentrically presented target will stimulate the nasal retina of one eye and the temporal retina of the fellow eye under binocular viewing conditions. Thus, any naso-temporal asymmetries in flicker detection will manifest as interocular differences in monocular FMTs that may adversely influence the BSR of flicker [8]. Such an effect is unlikely to influence the present study, for these stimuli were all presented at or close to the fovea where such naso-temporal asymmetries are potentially negligible (Fig 1).

In general, the quality of binocular vision depends on the similarity of the monocular inputs available for processing the cyclopean percept [19]. Stereoacuity [20] and binocular contrast summation in the spatial domain [10] are known to deteriorate with increasing interocular difference in the quality of the images presented to the two eyes. For instance, BSRs for contrast summation in the spatial domain reaches a value of unity–indicating no binocular summation–for anisometropic blur > 1.0 to 1.5D [21]. In the present study, the BSR’s of flicker remained similar for conditions with small and large interocular differences in monocular FMTs, indicating limited impact of the latter on binocular summation of flicker. These results are different from what is obtained in the spatial vision domain and must be interpreted with caution for two reasons. First, the instances of large interocular differences in monocular FMTs were limited (15 out of the total of 155 test runs) and could not be subject to robust statistical analysis. The BSR ranges reported here may thus be subject to change with alternations in the number of sample points. Second, the interocular differences in monocular FMTs were naturally occurring for unknown reasons and a significant fraction of them occurred for the smallest target size (7’) used in this study (Fig 7). This target was qualitatively adjudged by subjects to be the hardest amongst all test conditions and this condition also resulted in the largest intersession variability amongst all (Fig 2). The source of the interocular differences in monocular FMTs and the extent to which they are a reflection of task difficulty therefore remains unascertained in this study. Taken together, the relation between interocular differences in monocular FMTs and magnitude of BSR must be considered preliminary and needs confirmation in future studies that purposefully induce a range of interocular differences in monocular FMTs, as performed for contrast summation in spatial vision [22].

In conclusion, the visual system successfully summates monocular inputs from the two eyes to enhance flicker detection under binocular viewing conditions across a range of target sizes and luminance levels. The magnitude of this binocular advantage does not appear to depend on the absolute values of the corresponding monocular thresholds. These findings may serve as a predictive baseline for further experiments designed to determine how other stimulus properties and interocular differences in monocular thresholds may affect the binocular perception of flicker.

## Supporting information

S1 Data Two Microsoft excel spreadsheets containing the raw data of the study have been added as supporting information. The first spreadsheet–S1_datasheet–contains all the raw data and the second spreadsheet–S1_datasheet_WO–contains the data curated following the intersession variability analysis. A Matlab file–FMT_BSR_Analysis.m–that will plot the data of all the figures in the study using the two spreadsheets has also have been added as supporting information.
(ZIP)

S1 File (M)
